# Astaxanthin Treatment Confers Protection against Oxidative Stress in U937 Cells Stimulated with Lipopolysaccharide Reducing O_2_^−^ Production

**DOI:** 10.1371/journal.pone.0088359

**Published:** 2014-02-10

**Authors:** Sara Franceschelli, Mirko Pesce, Alessio Ferrone, Maria Anna De Lutiis, Antonia Patruno, Alfredo Grilli, Mario Felaco, Lorenza Speranza

**Affiliations:** 1 Department of Medicine and Science of Aging, University G. D’Annunzio, Chieti, Italy; 2 Department of Psychological, Humanistic and Territorial Sciences, University G. D’Annunzio, Chieti, Italy; University of Chieti, Italy

## Abstract

Recently, astaxanthin (ASTA) studies have focused on several biological functions such as radical scavenging, singlet oxygen quenching, anti-carcinogenesis, anti-diabetic, anti-obesity, anti-inflammatory, anti-melanogenesis, and immune enhancement activities. In this study, we investigated the potential role protective of ASTA, an antioxidant marine carotenoid, in restoring physiological conditions in U937 cells stimulated with LPS (10 µg/ml). Our results show that pre-treatment with ASTA (10 µM) for 1 h attenuates the LPS-induced toxicity and ROS production. The beneficial effect of ASTA is associated with a reduction intracellular O_2_^−^ production by restoring the antioxidant network activity of superoxide dismutase (SOD) and catalase (CAT), which influence HO-1 expression and activity by inhibiting nuclear translocation of Nrf2. We accordingly hypothesize that ASTA has therapeutic properties protecting U937 cells from LPS-induced inflammatory and oxidative stress.

## Introduction

Carotenoids are lipid-soluble pigments produced in some plants, algae, fungi and bacterial species which accounts for their red, orange, or yellow hues. Carotenoids are antioxidants due to their ability to quench singlet oxygen, be oxidized, and be isomerized [1]. In addition, carotenoids absorb light, hence providing defense from photo-oxidative damage. Astaxanthin (ASTA) is a red carotenoid pigment belonging to the Xanthophylls class of carotenoids. The physiological functions of ASTA have been studied for more than 20 years. It was found to have beneficial effects supporting human health and well-being and preventing pathologies. ASTA has been reported to be more effective than a representative carotenoid, β-carotene, for prevention of singlet oxygen quenching or generation, as well as lipid peroxidation in biological membranes. Moreover, it has been reported that some ASTA derivatives could scavenge superoxide anion radical [2]–[4].

The nuclear factor-erythroid 2-related factor-2 (Nrf2) anti-oxidant response element (ARE) pathway plays an important role in regulating anti-oxidant cellular defense. Nrf2-regulated cytoprotective genes include heme oxygenase-1 (HO-1), which has been shown to protect cells in models of inflammation, ischemia-reperfusion, hypoxia and hyperoxia-mediated injury [5], [6].

HO-1 is the inducible isoform of HO that catalyzes the first and rate-limiting step in heme degradation to produce equimolar quantities of biliverdin, carbon monoxide (CO) and free iron. Sequestration of free iron by ferritin lowers the prooxidant state of the cell [7], [8].

In addition to its role in heme catabolism, HO-1 has emerged as an important phase II and anti-inflammatory enzyme that is highly up-regulated by oxidative stress. Reactive oxygen species (ROS) are required for normal cellular homeostasis because they serve as critical mediators of cytokine signaling and antimicrobial host defenses. However, excessive ROS levels can induced cellular damage that compromises cellular survival [9], [10].

The aim of the present study was to assess the *in vitro* effect of carotenoid ASTA in a cellular inflammation model through evaluation of the antioxidant enzyme network (superoxide dismutase, SOD; catalase, CAT) and HO-1 expression and activity via Nrf2 on U937 cells stimulated with bacterial lipopolysaccharides (LPS).

## Materials and Methods

### Cell Culture

U937 mononuclear cells were purchased from American Type Culture Collection (Manassas, VA, USA). The cells were cultured in a 5% CO2 atmosphere in RPMI 1640 medium (GIBCO, Invitrogen) containing 10% fetal calf serum, 100 ng/mL streptomycin, 100 U/mL penicillin and 2 mM L-glutamine. Cells derived from the same freeze-down batch were thawed, grown and seeded (at 2×10^5^ cells per well) onto six-well tissue culture plates and cultured in medium with and without 10 µM astaxanthin (ASTA) (Sigma-Aldrich, St. Louis, MO, USA), LPS (10 µg/mL) and 10 µM of Protoporphyrin IX zinc (ZnPP IX)(Sigma-Aldrich, St. Louis, MO, USA). ASTA was dissolved in dimethyl sulfoxide (DMSO) and diluted with the medium. The final concentration of DMSO in the medium was 0.5%. Control cells did not contain ASTA and/or LPS.

### Cell Viability Assay

MTT, absorbed into the cell and eventually the mitochondria, is broken down into formazan by mitochondria succinate dehydrogenase. Accumulation of formazan reflects the activity of mitochondria directly and cell viability indirectly. Cell viability was measured by the MTT assay. U937 cells were seeded on 96-well plates at a density of 8×10^3^ cells/well, cultured and treated according to the method described above. A total of 20 µl of MTT was added at a concentration of 0.5 mg/ml after medium (200 µl) was added to each well. The plates were incubated at 37°C for 4 h to dissolve the formazan that had formed. The solution (220 µl) was removed from each well and 150 µl of DMSO was added. Reduced MTT was measured on an ELISA reader (Bio-Rad, Hercules, CA, USA) at a wavelength of 570 nm. Values are expressed as a percentage of the control value.

### Western Blot Analysis

Total protein extracts were prepared by treating cells with lysis buffer (RIPA). Nuclear extracts were prepared as previously described [11].

Proteins were quantified using the Bradford method. Western blot analysis was performed as described previously [12] using the following primary antibodies: anti-Nrf2 (Santa Cruz Biotechnology, Santa Cruz, CA, USA) 1∶200 and anti-HO-1 (Stressgen, Ann Arbor, MI, USA) 1∶1000.

Nitrocellulose was then washed in TBS, and incubated with secondary antibody HRP-conjugated (dilution 1∶10000, Pierce) for 1 h, washed again and developed. The nitrocellulose was scanned using a computerized densitometric system (Bio-Rad Gel Doc 1000, Milan, Italy). Protein levels were normalized to the housekeeping protein actin and tubulin (Sigma-Aldrich) to adjust for variability of protein loading, and expressed as a percentage of vehicle control.

### Quantization of Intracellular Superoxide Production using a Colorimetric NBT Assay

Nitro blue tetrazolium (NBT) (Sigma-Aldrich) assay, performed according to the method described by ROOK et al. (1985), was used to evaluate O_2_^−^ production. Briefly, after each treatment, cells were incubated with 0.1 mg/ml NBT in pre-filtered culture medium for 3 hours at 37°C; they were then washed three times with methanol. The amount of NBT-formazan produced is an indicator of intracellular production of superoxide anion and can be determined spectrophotometrically (SpectraMax® 190, Molecular Devices) at 630 nm after solubilization of crystals in 200 µl of KOH 2M/DMSO solution.

### Cu, Zn-Superoxide Dismutase Activity

SOD activity was determined as described previously [13]. The assay mixture contained 50 mM sodium carbonate buffer, pH 10, epinephrine 0.1 mM (Sigma-Aldrich), and tissue fraction (containing about 1–50 µg of protein) in a final volume of 2.5 mL. The inhibitory effect of SOD on the autoxidation of epinephrine, by use of 1.25 mM KCN to discriminate CN- insensitive MnSOD from CN-sensitive CuZnSOD, was assayed spectrophotometrically at 480 nm at 25°C. Percentage inhibition values were converted into activities by using a purified Cu, Zn bovine SOD as standard (Sigma-Aldrich). One unit of SOD is the amount of enzyme required to halve the rate of substrate auto-oxidation.

### Catalase Activity

CAT activity was measured spectrophotometrically as described previously [14]. The decomposition of H_2_O_2_ was monitored continuously at 240 nm. The assay mixture in a final volume of 3 mL contained 10 mM potassium phosphate buffer, 10 mM H_2_O_2_ and 1.5–11 µg of protein of enzymatic extract. CAT units were defined as 1 µmole H_2_O_2_ decomposed/min at 25°C.

### Assay for HO Activity

An HO activity assay was performed as described previously [7]. Briefly, microsomes from harvested cells were added to a reaction mixture containing 0.8 mM NADPH (Sigma-Aldrich), 2 mM glucose 6-phosphate (Sigma-Aldrich), 0.2 U glucose-6-phosphate dehydrogenase (Sigma-Aldrich), 2 mg rat liver cytosol prepared a 105000 g from supernatant fraction as a source of biliverdin reductase, potassium phosphate buffer (100 mM, pH 7.4) and 20 mM hemin (Sigma-Aldrich).

The reaction was conducted at 37°C in the dark for 1 h and then placed on ice for 2 min to terminate the reaction. Bilirubin was determined by calculation from the difference in absorbance between 464 and 530 nm (extinction coefficient, 40 mM^−1^ cm^−1^ for bilirubin). HO activity was expressed as nanomoles of bilirubin formed per milligram of microsomal protein per hour. The total protein content of confluent cells was determined using a Bio-Rad DC protein assay (Bio-Rad, Herts, UK) by comparison with a standard curve obtained with bovine serum albumin.

### Statistical Analysis

The results are expressed as means ± SD or mean ± SEM. Statistical analysis was performed using analysis of variance (ANOVA). A null hypothesis probability of <5% (*p*<0.05) was considered statistically significant. The comparison LPS *vs.* LPS+ ASTA was performed using a post hoc test with the alpha level at 0.05.

## Results and Discussion

### Effects of LPS and Astaxanthin on Cell Viability

Macrophage activation by bacterial LPS is an important event involved in inflammation and inflammation-related diseases. LPS-induced macrophage activation is characterized by the induction or up-regulation of genes such as nuclear factor-kappaB (NF-κB), activator protein-1 (AP-1) and cytokines, which play a crucial role in the inflammatory process [14]–[16].

**Figure 1:**
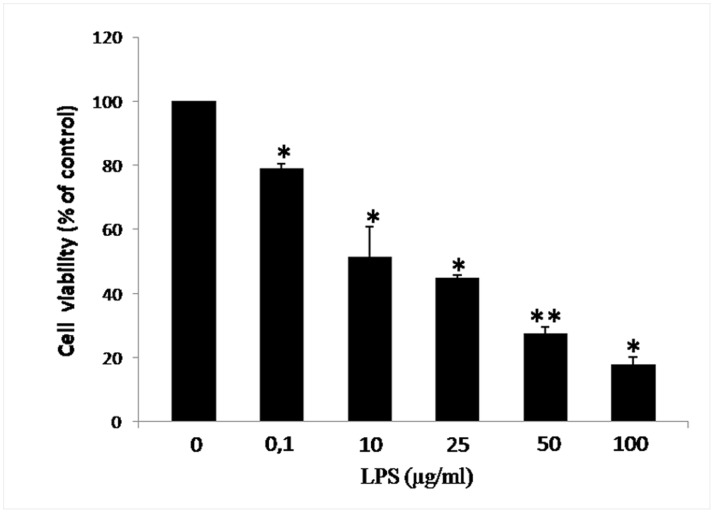
Influence of LPS on cell viability. U937 cell were exposed to 10 µg/mL of LPS for 24 h and cell viability was assessed by MTT assay. Results are expressed in percentage of control (mean ± SEM of five independent experiments, performed in triplicate). *p<0.05 vs control cells (0 µg/mL of LPS); **p<0.01 vs control cells.

To evaluate the viability of U937 cells after exposure to inflammatory injury, cells were treated with different concentrations of LPS (0.1–100 µg/mL) for 24 h. After washing, and following 24 h incubation, cell viability was measured using the MTT assay. LPS significantly decreased MTT levels, indicating a lower cell count, in a concentration-dependent manner (Figure 1). Treatment with a concentration of 10 µg/mL of LPS for 24 h was selected for subsequent experiments as it reduced cell viability to approximately 43±9.2% of control (*p<0.05, Figure 1).

**Figure 2:**
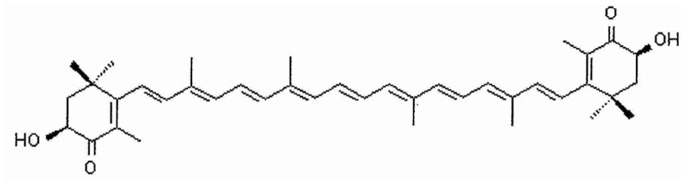
Chemical structure of ASTA.

In a previous work we evaluated the effect of ASTA (Figure 2) on cell viability in the U937 cell line. An MTT reduction experiment showed that ASTA did not exhibit any cytotoxic effects on U937 cells at concentrations of 10 µM [16]. Hence, for all experiments this concentration of ASTA was employed.

**Figure 3:**
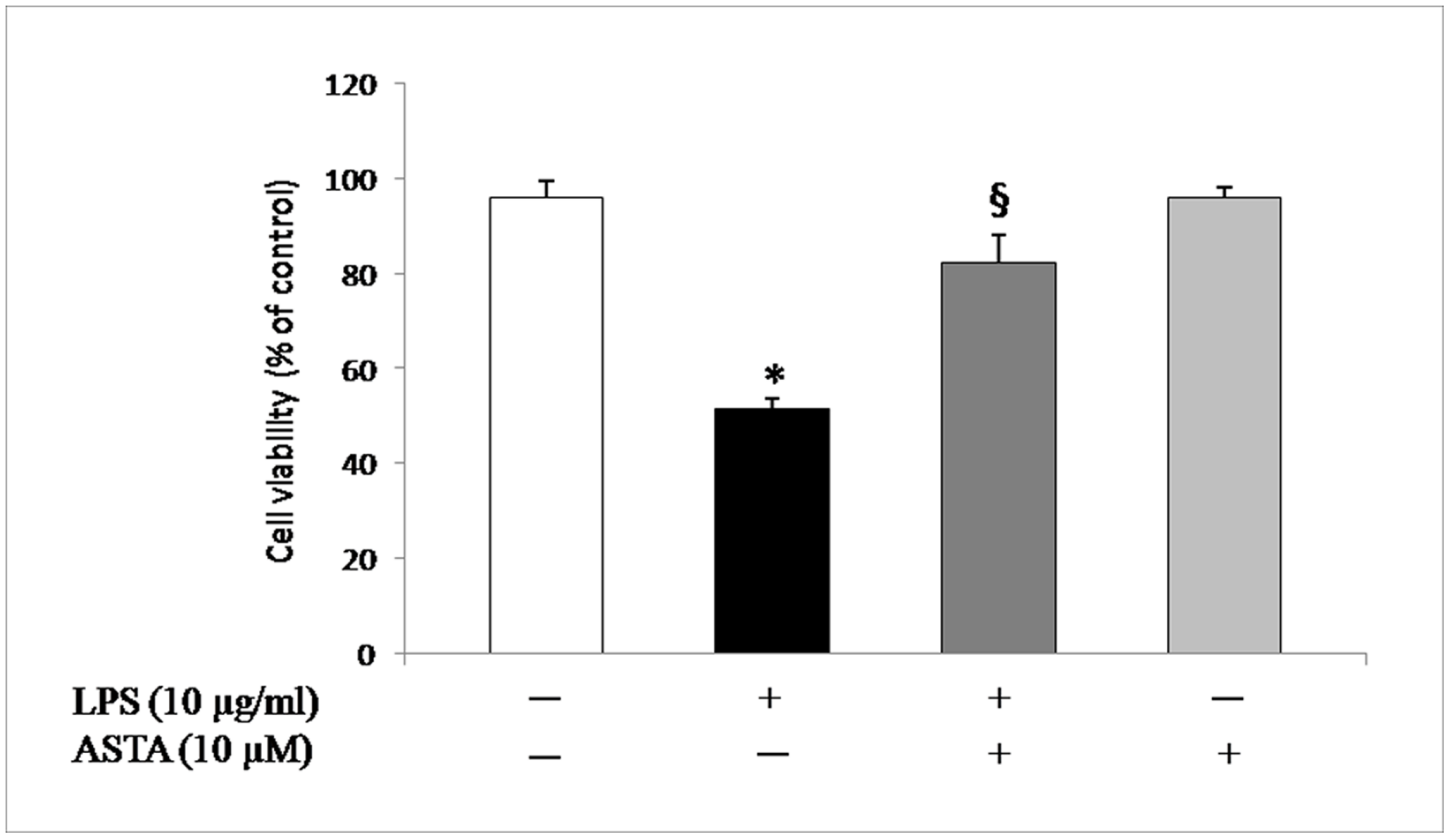
Effects of ASTA on viability of U937 cells. Cells were incubated with or without ASTA (10 µM) for 1 h, and then stimulated with LPS (10 µg/ml) for another 24 h. Cell viability was determined as described in the Materials and Methods section. Data are the mean values of three experiments ± SEM. *p<0.05 vs. control cells; ^§^p<0.05 vs. LPS or ASTA treated cells.

In the U937 cell line, the viability of LPS-treated cells decreased significantly, but that of cells that were pre-treated for 1 h with ASTA at a concentration of 10 µM, prior to exposure to LPS for 24 h, increased significantly to 82.3±5.9% (^§^p<0.01) (Figure 3). Consequently ASTA exerted a protective effect against LPS-induced cytotoxicity.

### Effect of Astaxanthin on LPS-induced Oxidative Stress

During the inflammatory process, high amounts of reactive oxygen species (ROS), such as superoxide anion (O_2_^−^), hydrogen peroxide (H_2_O_2_) and hydroxyl radical (OH^.^), contribute to intracellular destructive mechanisms [14], [17].

To assess the antioxidant property of ASTA against LPS-induced inflammation in U937 cells, ROS generation and oxidative stress, an NBT assay was carried out. This method is usually used to detect possible involvement of reactive oxygen species (ROS), primarily superoxide anion radical O_2_^−^, in the process of toxicity and cell death [18].

**Figure 4:**
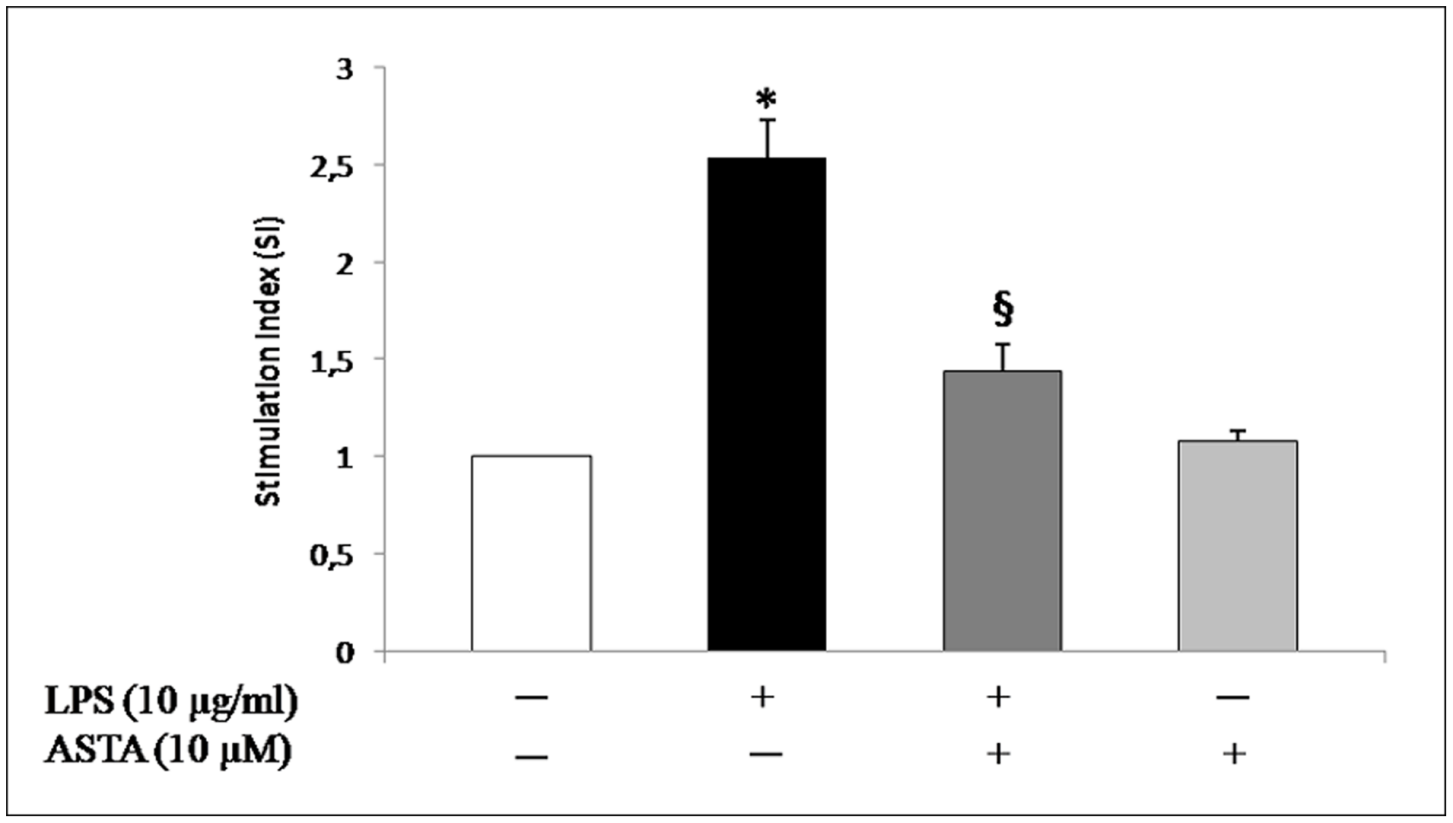
Effect of ASTA treatment on superoxide anion generation assessed by estimating reduced nitrobluetetrazolium (NBT) inU937 cells. O_2_^−^ production increased significantly in U937 cells stimulated with LPS. ASTA blocked LPS-induced O_2_^−^ production. Values are means±SD of values from three experiments. The control value (no addition of ASTA) was set at 1. *p<0.05 vs control cells; ^§^p<0.05 vs. LPS or ASTA treated cells.

The NBT reduction assay is shown in Figure 4. The experiments were performed in triplicate. The results were expressed as an NBT reduction stimulation index (SI) which was calculated as the optical density (OD) ratio of treated and control cells. The stimulation index (SI) for the control was taken to be 1.

Treatment of U937 cells with LPS (positive control) showed significant (*p<0.05) stimulation of NBT reduction compared to control cells.

Free radicals are potentially toxic; they are usually inactivated or scavenged by antioxidants before they can inflict damage on lipids, proteins or nucleic acids. To ameliorate and cope with injury from oxidative damage and maintain redox homeostasis, aerobic organisms have a complex antioxidant defense system that includes antioxidant enzymes such as superoxide dismutase (SOD) and catalase (CAT). These block the initiation of free radical chain reactions [19], [20].

**Figure 5:**
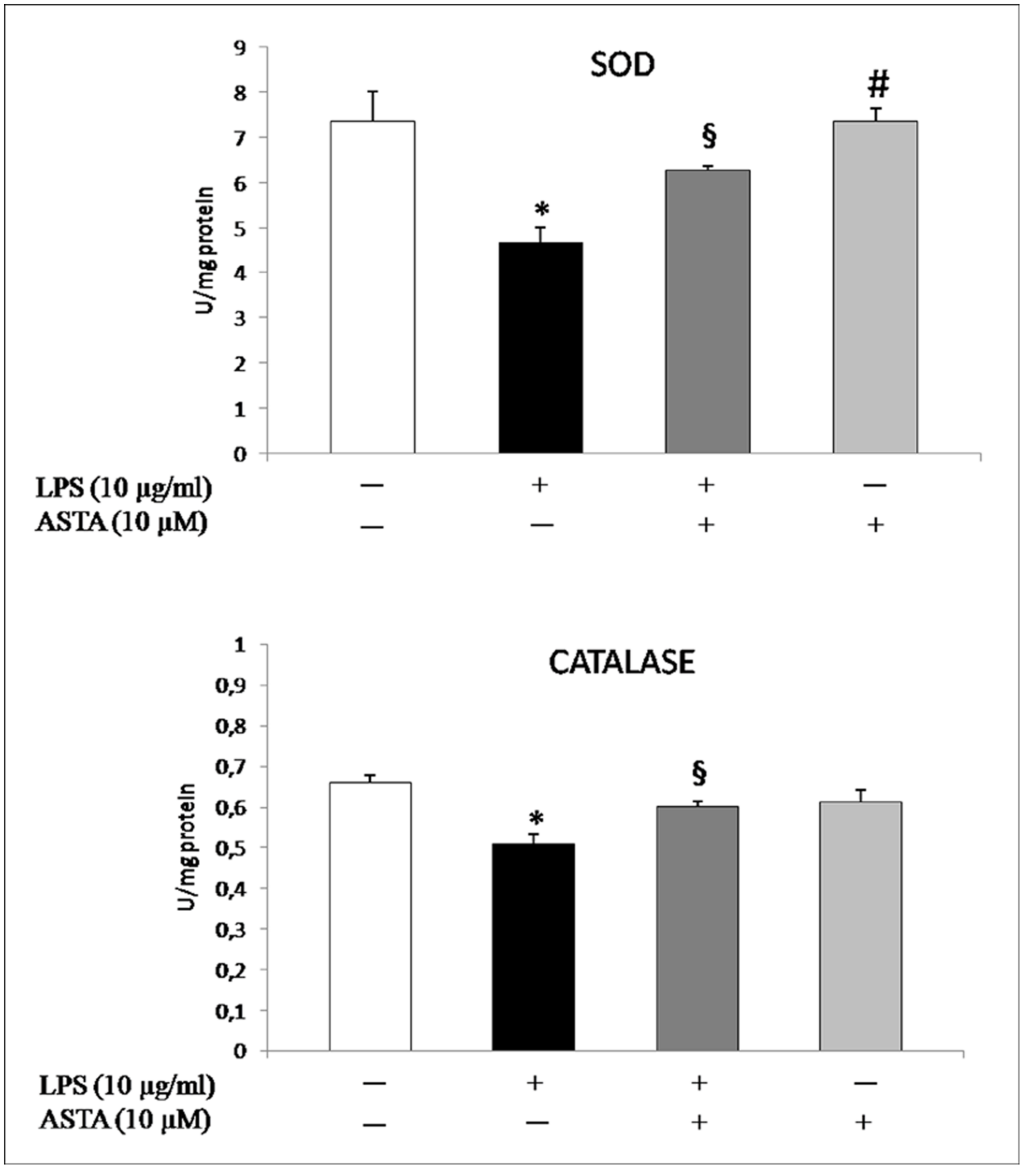
Effect of ASTA on antioxidant enzymes, SOD and CAT, in U937 cells. The activities of SOD and CAT were measured as described in Materials and Methods. Cells were incubated with the indicated concentrations of either or both ASTA and LPS. Results presented are means ± SD (*n* = 3) and have been statistically analyzed. *p<0.05 vs. control cells; ^§^p<0.05 vs. LPS treated cells; # p<0.05 vs LPS +ASTA treated cells.

In LPS-stimulated U937 cells there is a high level of O_2_^−^ showing that under similar conditions of elevated levels of oxidative stress the dismutase reaction of SOD is reduced (Figure 5).

This decrease in SOD activity is associated with a parallel decline in CAT activity, with consequent disregulation of the antioxidant enzymatic system and accumulation of O_2_^−^ which is potentially harmful to the cell. Excessive redox-active species may repress the activity of cellular enzymes and activate transcriptional factors involved in resolution of the inflammatory process [21], [22].

Treatment with ASTA 10 µM shows no significant (^§^p<0.05) stimulation of NBT reduction, due to a lower production of O_2_^−^ which, as shown in Figure 4, appears to be comparable to baseline values. This marked decrease in oxidative stress is combines with reactivation of the detoxification mechanism attributable to CAT and SOD activity being restored by ASTA-treatment (Figure 5).

Our finding is thus that ASTA exerted a powerful antioxidant effect on O_2_^−^ produced by the LPS-stimulated U937 cell line.

### Involvement of the Nrf2/HO-1 System in the Antioxidant Effects of Astaxanthin

We have previously shown that ASTA blocks H_2_O_2_ mediated activation of NF-κB and its downstream cytokine secretion through modulating protein tyrosine phosphatase-1 (SHP-1) expression [16].

Activation of NF-κB, a transcription factor, controls the expression of several inducible genes such as HO-1 and iNOS engaged in the inflammatory response and involved in cellular defense against oxidative stress. A number of proinflammatory agents are potent activators of the HO-1 response. The most notable example is bacterial LPS, which typically produces robust HO-1 activation in cell cultures [23], [24].

It has, in fact, been shown that the expression of HO-1 is crucial in inhibiting LPS-induced proinflammmatory responses in RAW 264.7 cells and that ROS mediates the induction of HO-1 in a redox-regulated pathway [23], [25].

HO-1 is a well-known Nrf2 target gene with established anti-oxidative and anti-inflammatory properties. Nrf2 plays an important role in inducing HO-1 in many cells, including macrophages [26].

Under normal homeostatic conditions, Nrf2 is retained in the cytoplasm via its interaction with Kelch-like ECH-associated protein 1 (Keap1) and is degraded by the proteasome. Under oxidative conditions, Nrf2 is released from Keap1, translocates to the nucleus, recognizes and binds to the ARE site, which causes transcription of its target genes [27].

**Figure 6:**
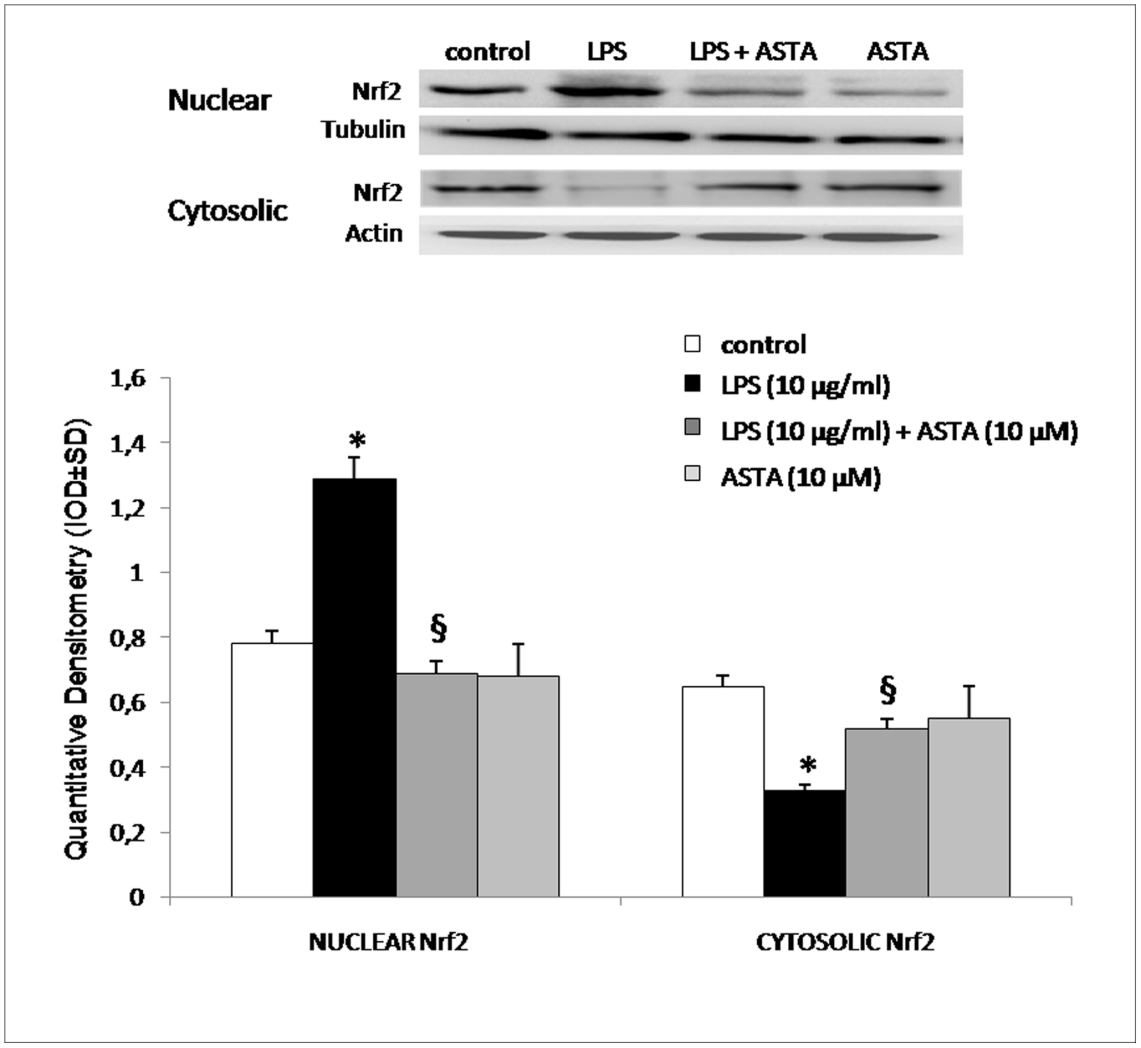
Effect of ASTA on expression of Nrf2. U937 cells were treated with LPS or ASTA at the doses indicated. Groups include the following: Nuclear, Nrf2 protein levels in nuclear extracts; Cytosolic, Nrf2 protein levels in cytosolic fractions; panels on left show results of Western blot as appropriate. Plot on right shows the results of quantitative analysis. *p<0.05 compared with control cells; ^§^ *p*<0.05 compared with LPS-treated cells.

We thus needed to determine whether or not the O_2_^−^ reduction observed in U937 cells treated with ASTA reduces Nrf2 nuclear translocation. For these experiments we prepared nuclear extracts and cytosolic fractions, and measured Nrf2 levels in each fraction by Western blot analysis. As shown in Figure 6, Nrf2 was retained in the cytosolic fraction in untreated cells, whereas it was translocated to the nuclear fraction when treated with LPS.

In such a condition, nuclear Nrf2 expression is up-regulated, playing an important role in regulating cellular defense through induction of HO-1 expression [28], [29].

The induction of HO-1 may therefore be a generalized marker of oxidative stress, for which reason we measured the effects of ASTA treatment on alteration of the cellular redox state and hence on HO-1 protein.

**Figure 7:**
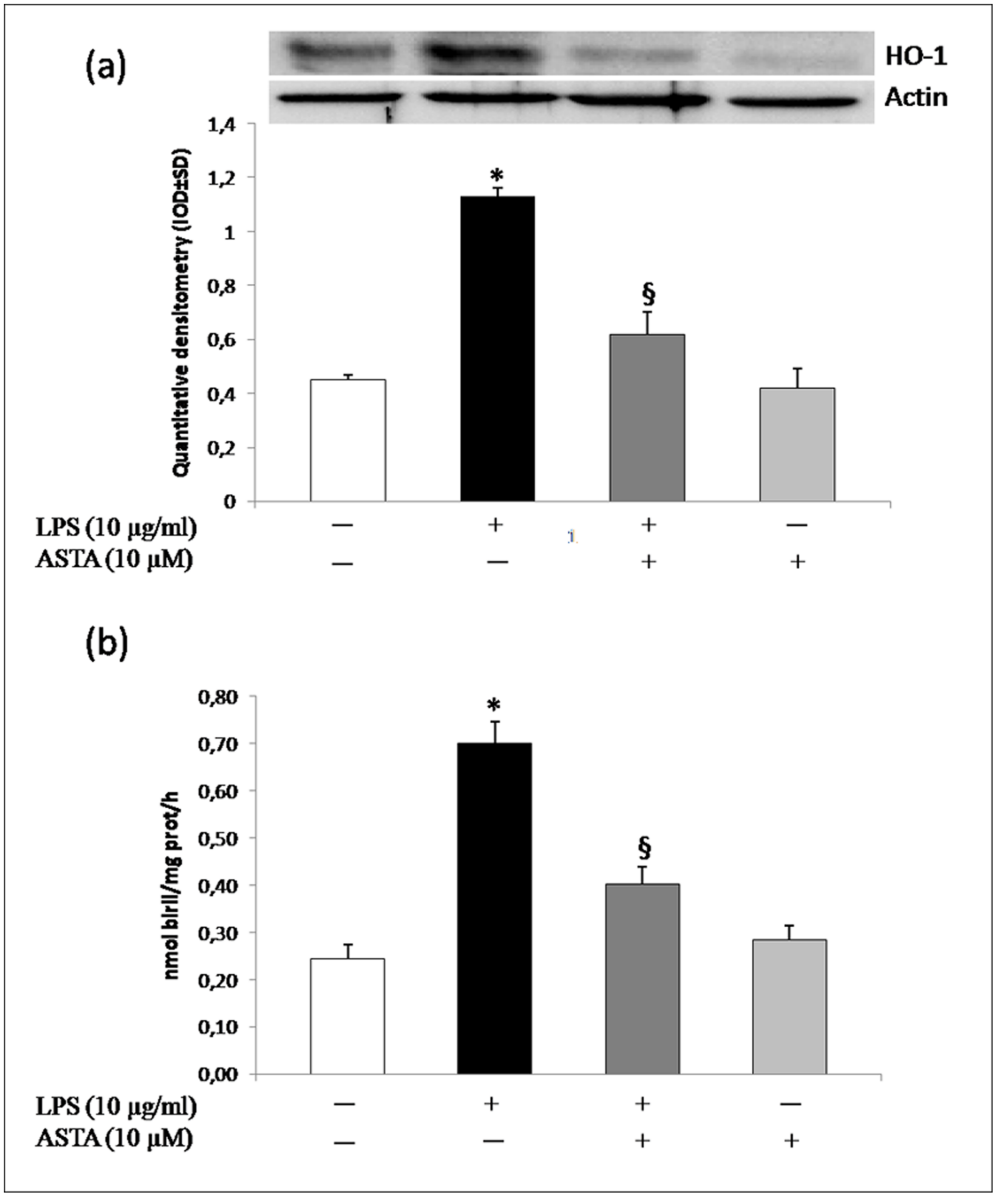
Effect of ASTA on expression and activity of HO-1. A) Western blot and quantitative analysis for HO-1 expression, actin being used as loading control; data shown are means ± SD, *p<0.05 compared with control cells; ^§^ *p*<0.05 compared with LPS or ASTA treated cells; data were from at least three independent experiments, each performed in triplicate. B) Mean HO activity (nmol/bilirubin/mg protein)/h ± SD, n = 3) detectable in homogenates obtained from U937 cells: untreated; treated with LPS, ASTA and LPS+ASTA. *p<0.05 compared with control cells; ^§^ *p*<0.05 compared with LPS or ASTA treated cells.

**Figure 8:**
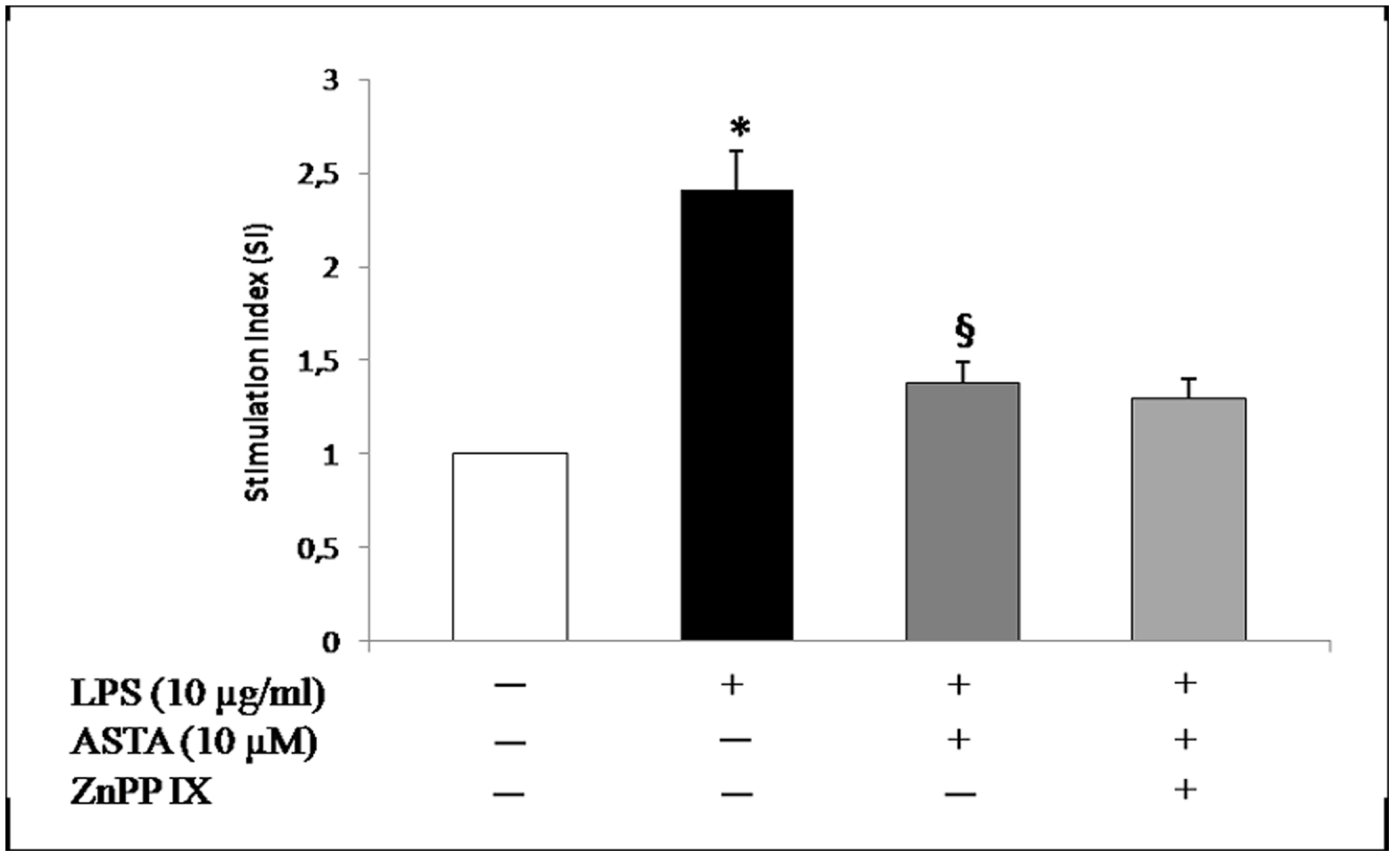
The effect of HO-1 inhibition and ASTA treatment on cellular superoxide anion release. Values are means±SD of values from three experiments. The control value (no addition of ASTA) was set at 1. *p<0.05 vs control cells; ^§^p<0.05 vs. LPS treated cells.

HO-1 analysis (Figure 7) showed that, when the cells were stimulated with LPS, ASTA induced a return a the physiological state, decreasing O_2_^−^production, restoring scavenger enzyme activity (SOD and CAT), and hence reducing the expression and activity of HO-1 protein (Figure 7A,B). To support our hypothesis, Protoporphyrin IX zinc (ZnPPIX), an HO inhibitor, was used. As shown in Figure 8, O_2_^−^ reduction is associated only with ASTA treatment. This meant that ASTA was able to restore the cellular redox state by reduction of O_2_^−^ production, mediated by resumption of SOD and CAT activity, which preventsNrf2 translocation in U937 cells, resulting in down-regulation of the expression of inducible genes such as HO-1.

## Conclusions

Oxidative stress caused by the imbalance between reactive oxygen species (ROS) and biological antioxidant systems can lead to modification of macromolecules such as DNA, lipids, and proteins. Upon exposure to cellular stress this physiological homeostasis is endangered. It is known that the induction of HO-1 can be a response to and a generalized marker of oxidative stress [30].

We have demonstrated that the marine drug ASTA exerts an antioxidant effect by suppressing the O_2_^−^ level in an LPS-stimulated U937 cell line. This occurs through the ability of ASTA to interact with various different radicals: the pattern of conjugated double bonds in the polyene backbone of ASTA affects its quenching of singlet oxygen [31]. Quenching involves the transfer of excitation energy from O_2_^−^ to the carotenoid, resulting in ground state oxygen and an excited triplet state carotenoid. The energy is dissipated between the excited carotenoid and the surrounding solvent to yield ground state carotenoid and thermal energy. In the process of quenching the carotenoid remains intact and can undergo further cycles of singlet oxygen quenching [32].

In lowering the O_2_^−^ levels, treatment with ASTA, therefore, leads to functional recovery of the antioxidant network (SOD and CAT). Consequently, as shown by our results, the nuclear translocation of Nrf2 does not occur, and neither does any parallel up-regulation of the expression and catalytic activity of HO-1.

The interest of our research lies in the current need to find new substances of natural origin which have proven efficacy and a low degree of toxicity in the therapeutic treatment of various diseases characterized by alteration of the cellular redox state.

From the results gathered the conclusion is that the natural compound ASTA may be used for therapeutic purposes to protect cells against oxidative conditions.
